# Volatile Analysis of Wuliangye Baijiu by LiChrolut EN SPE Fractionation Coupled with Comprehensive GC×GC-TOFMS

**DOI:** 10.3390/molecules27041318

**Published:** 2022-02-15

**Authors:** Jia Zheng, Zhanglan He, Kangzhuo Yang, Zhipeng Liu, Dong Zhao, Michael C. Qian

**Affiliations:** 1 Flavor Science Innovation Center, Technology Research Center, Wuliangye Yibin Co., Ltd., 150# Minjiang West Road, Cuiping District, Yibin 644000, China; hezhanglan@wuliangye.com.cn (Z.H.); yangkangzhuo@wuliangye.com.cn (K.Y.); liuzhipeng@wuliangye.com.cn (Z.L.); zhaodong@wuliangye.com.cn (D.Z.); 2 Department of Food Science and Technology, Oregon State University, Corvallis, OR 97331, USA

**Keywords:** baijiu, volatile fractionation, Wuliangye, LiChrolut^®^ EN SPE, GC×GC-TOFMS

## Abstract

Wuliangye baijiu is one of the most famous Chinese liquors with a protected geographical indication. This study used LiChrolut^®^ EN-based solid-phase extraction (SPE) and fractionation combined with comprehensive two-dimensional chromatography-time-of-flight mass spectrometry (GC×GC-TOFMS) to unveil its volatile composition. The volatiles were isolated with LiChrolut^®^ EN-based SPE and traditional liquid-liquid extraction (LLE). The neutral/basic fractions from LLE and the SPE were fractionated on a LiChrolut^®^ EN SPE column and analyzed by comprehensive GC×GC-TOFMS. Compared with LLE, more esters and alcohols were detected in the SPE-based extraction. The SPE fractionation and GC×GC-TOFMS analysis resulted in the identification of about 500 volatile compounds in more than 3000 peaks of the Wuliangye baijiu. The approach simplifies the complex baijiu composition into functional group-based fractions for reliable identification and analysis. This study provided a confidence volatile identification approach for Chinese baijiu based on the SPE fractionation GC×GC-TOFMS.

## 1. Introduction

Chinese liquor (baijiu) is a complex alcoholic beverage that contains almost all classes of volatile compounds such as esters, acids, alcohols, ketones, aldehydes, acetals, phenolic compounds, sulfur-containing compounds, and others [1]. Meanwhile, esters, alcohols, and acids are the main compounds in most Chinese baijiu, especially the strong aroma type baijiu [2,3]. Due to its complexity, the conventional gas chromatography-mass spectrometry (GC-MS) technique cannot analyze all compounds effectively due to coelution on a single GC column. Therefore, comprehensive pre-separations before GC-MS analysis are often needed to reveal the composition of baijiu.

Volatile compounds in alcoholic beverages can be extracted using liquid-liquid extraction (LLE), solid-phase extraction (SPE), solid-phase microextraction (SPME), stir bar sorptive extraction (SBSE), and a few others. The LLE approach is a classical volatile isolation method widely used in food and beverage. However, after extraction, further purification steps such as solvent-assisted flavor evaporation [4] are often needed to remove nonvolatile components. The SPE approach is another widely used method that integrates sample extraction, concentration, and purification in one SPE cartridge [5]. It has several advantages: simple operation, less solvent consumption, easy standardization, and good repeatability. LiChrolut^®^ EN sorbent, a commercial hydrophobic polymer—ethylvinyl benzene-divinylbenzene [6], presents excellent extraction ability to adsorb a wide range of volatile compounds in various foodstuffs as well as alcoholic beverages [7].

Fractionation is an effective technique to divide the complex composition into several fractions according to their chemical properties, such as polarity, pKa, and functional groups [8]. Based on pH and salt modification, neutral/basic, acidic, and water-soluble compounds could successfully be separated based on liquid-liquid fractionation [9]. Normal phase chromatography based on silica gel is a common approach for achieving fractionation based on polarity [10]. Meanwhile, some new approaches were developed recently to analyze volatile compounds in Chinese baijiu. He et al. [11] provided a novel method using tandem SPE columns of LiChrolut^®^ EN and silica gel to achieve simultaneous volatiles’ extraction and fractionation, successfully eliminating the esters’ interference. However, silica gel has active sites and could induce catalytical conversion of the analytes; it is desirable to use LiChrolut^®^ EN SPE for fractionation as an alternative for silica gel.

Comprehensive two-dimensional chromatography with time-of-flight mass spectrometry (GC×GC-TOFMS) is an ideal approach that separates complex volatile compounds in the GC×GC system through two orthogonal columns (boiling point or polarity). It identifies compounds based on acquired accurate mass by TOF mass spectrometer [12] and retention indices on the GC column. GC×GC-TOFMS has been used to study volatile composition in Chinese baijiu [13] and discriminate baijiu from different origins and types [14,15,16]. Wang et al. [17] used this technique to compare the extraction ability of three common pretreatment methods on the Chinese baijiu, and the results showed that different pretreatment methods were beneficial for different types of volatile compounds.

Wuliangye baijiu is one of the most premium distilled liquor brands in China. It also represents a high-end quality grade in the Chinese baijiu industry because of its much-appreaciated aroma and taste features. In 2019, Wuliangye baijiu was listed in geographical indication protected products in the China–European Union bilateral agreement [18]. Wuliangye baijiu is fermented from five species of grains, including sorghum, rice, glutenous rice, wheat, and corn [19].

In 2006, Fan and Qian [20] firstly used GC-olfactometry (GC-O) technology to analyze Wuliangye after extracting the aroma compounds by LLE and fractionating to acidic/water-souble, basic, and neutral fractions; a total of 126 aroma compounds were identified, mainly including esters, pyrazines, and furans. Niu et al. [21] compared the aroma compounds in three Wuliangye of different ages by HS-SPME coupled with GC-O; the results revealed that nine aroma compounds could be regarded as the key aroma compounds based on their flavor dilution (FD) values, and the total content of aroma compounds decreased with age. He et al. [11] attempted an approach to achieve simultaneous aroma extraction and fractionation using tandem LiChrolut^®^ EN and silica gel SPE columns; volatiles were separated to different fractions based on their respective polarities. However, the detailed chemical makeup of the Wuliangye baijiu aroma is still not fully understood.

## 2. Results and Discussion

### 2.1. GC×GC-TOFMS Performance

**Figure 1:**
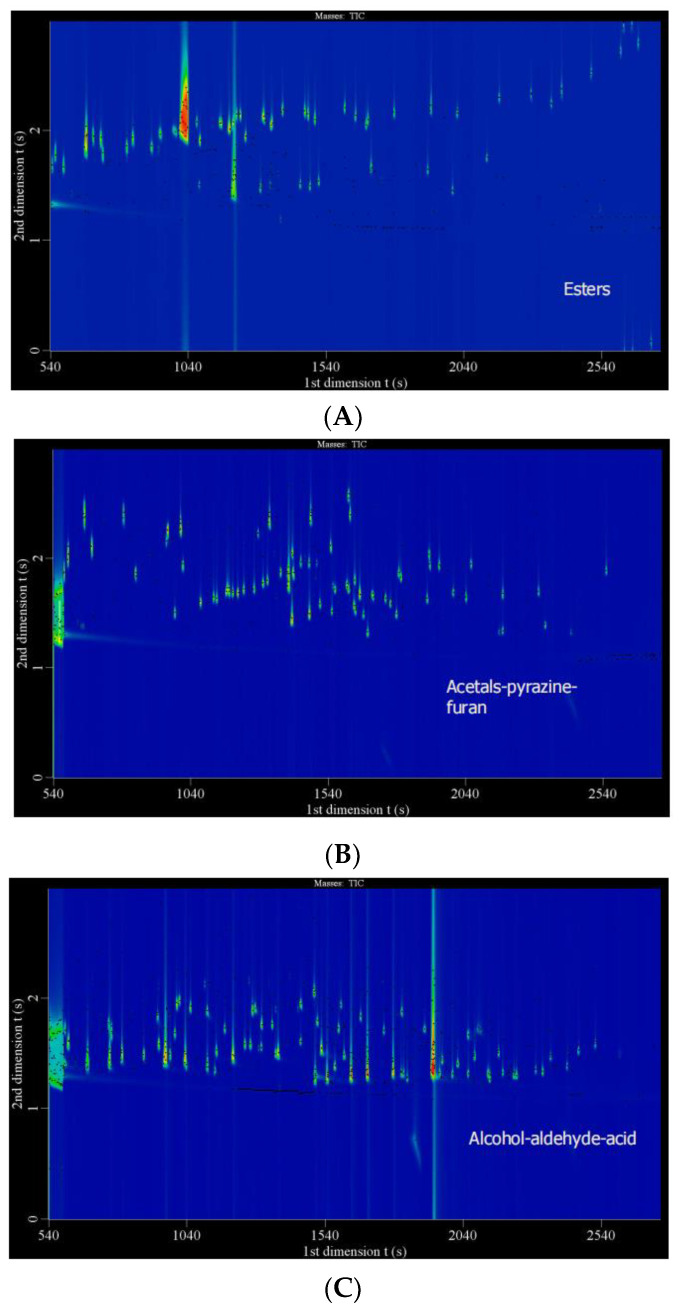
Authentic chemical standards for testing the applicability of GC×GC-TOFMS system. (**A**). Esters; (**B**). Mixture of acetals, pyrazines, and furans; (**C**). Mixture of alcohols, aldehydes, and acids.

Two-dimensional GC has a better separate ability and higher resolution than the conventional one-dimensional GC, so it is ideal for complex sample analysis. To confirm the feasibility and accuracy of comprehensive two-dimensional chromatography for compounds identification, the testing system of GC×GC-TOFMS was evaluated using a total of three groups of authentic chemical standard mixtures (about 400 compounds in 1 mg/L) known in baijiu (Figure 1). Most compounds were detected and identified with similarity and reverse similarity of more than 800 in both cases (Figure 1). Therefore, it suggested that the testing system was suitable for compounds identification.

**Figure 2:**
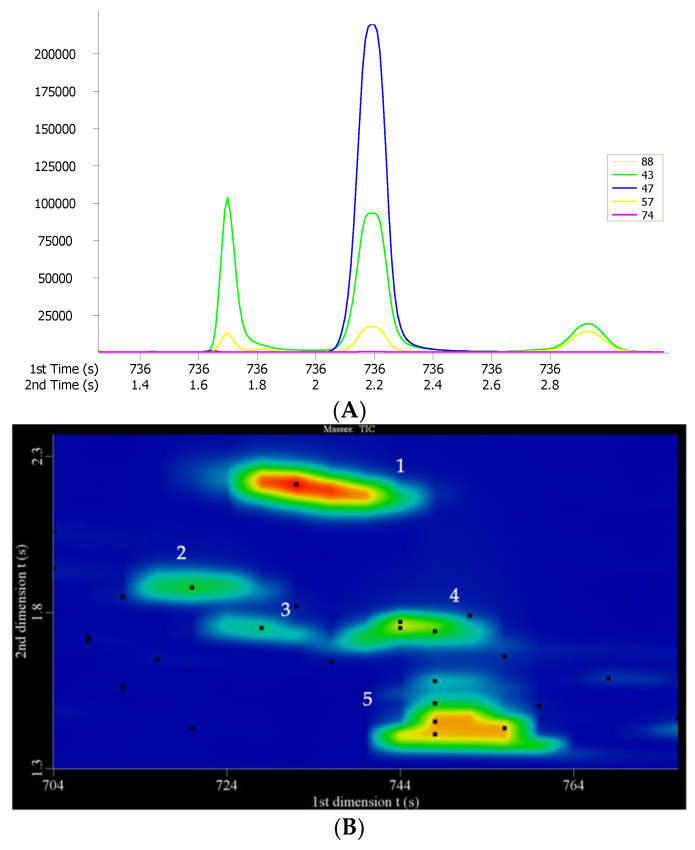
(**A**) Four peaks shown in the conventional chromatogram. (**B**) Five peaks clearly separated in the contour chromatogram. 1. 1,1-diethoxy-3-methylbutane (*m*/*z* 47), 2. ethyl 3-methylbutanoate (*m*/*z* 88), 3. butyl acetate (*m*/*z* 43), 4. hexanal (*m*/*z* 57), and 5. 2-methylpropanol (*m*/*z* 74).

Figure 2 demonstrated the effectiveness of resolution of ^2^D for coeluting compounds in ^1^D GC. For example, 1,1-diethxoybutane and ethyl acetate were coeluted in the ^1^D column but successfully separated in the ^2^D column. Furthermore, after automated spectral deconvolution process, the coeluted compounds including 1,1-diethoxy-3-methylbutane (*m*/*z* 47), ethyl 3-methylbutanoate (*m*/*z* 88), and butyl acetate (*m*/*z* 43), were also successfully identified.

**Figure 3:**
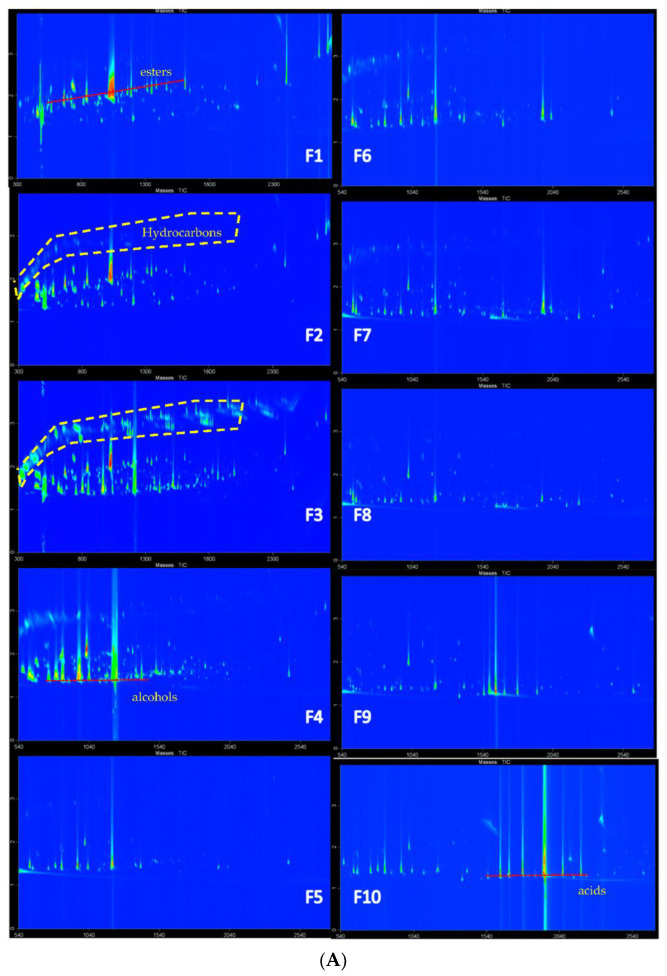

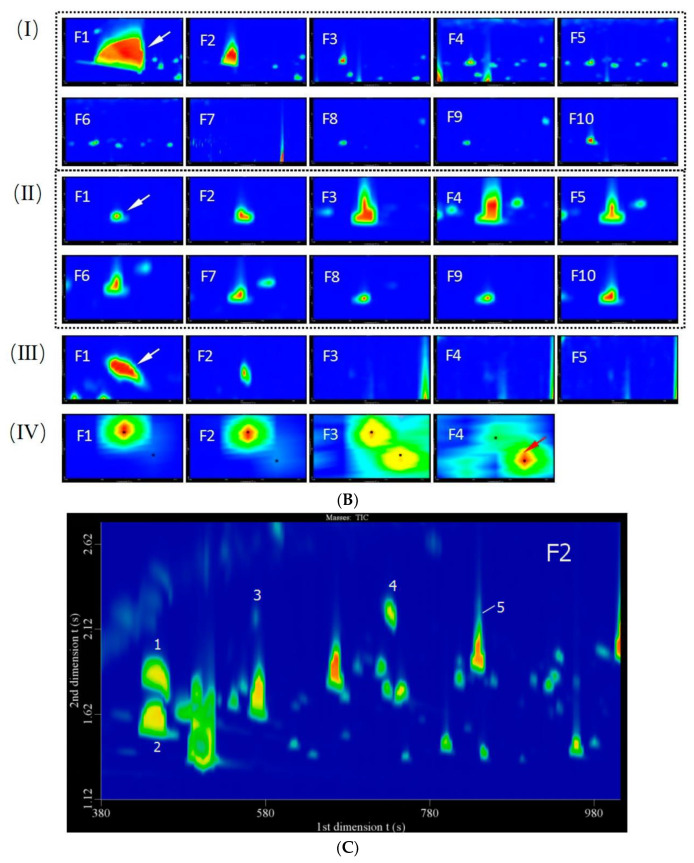
(**A**) GC×GC total ion chromatogram contour plots of fractions from Fraction 1 to Fraction 10 from SPE extract. First dimension time range: 300–2700 s, and second dimension time range: 0–4 s. (**B**) Distribution of (I) ethyl hexanoate, (II) furfural, (III) 1,1-diethoxy-3-methylbutane, and (IV) β-damascenone in fractionation. (**C**). Main acetals in fraction 2 of Wuliangye baijiu. 1. 1,1-diethoxyethane, 2. ethyl acetate, 3. 1,1-diethoxybutane, 4. 1,1-diethoxy-3-methylbutane, 5. 1,1-diethoxypentane.

Previously, the silica gel normal phase chromatography successfully separated volatile compounds in Chinese Yanghe baijiu based on functional groups and molecular polarities [10]. However, silica gel-based fractionation could induce catalytical conversation of compounds due to active sites on silica gel. In this study, a hydrophobic LiChrolut^®^ EN-based SPE was employed to separate the volatile extracts into multiple fractions according to their polarities. As shown in Figure 3A, a good separation of volatile compounds with different polarities is achieved. Based on polarity, esters were first eluted out, followed by alcohols and carboxy acids, being the most polar, presented in the last two fractions. A few compounds, however, were distributed into multiple fractions (Figure 3B), due to high concentrations or high polarities. As shown in Figure 3B, abundances of ethyl hexanoate and 1,1-diethoxy-3-methylbutane decrease with the increase of polarity of mixed elution solvents. Furfural was enriched in F3 and F4. *β*-Damascenone was identified in F2 and F3.

### 2.2. Number of Volatile Compounds

In this study, automated processing of GC×GC-TOFMS data was applied to tentatively identify all peaks in the GC×GC chromatogram contour plots with a S/N threshold greater than 20. An RI deviation between RI and retention index in published literature (RIL) with no more than 30 was considered applicable for compound determination [22]. Compounds identification was further confirmed with authentic standards in lab. From both LLE and SPE extraction, a total of more than 3000 peaks was found, and a total of 666 compounds was determined, in which 423 compounds were in common (63.5%); 95 compounds were only detected in LLE-based extraction (14.3%), and 148 compounds were only detected in SPE-based extraction (22.2%).

### 2.3. Distribution of Volatile Compounds in Fractionation

**Figure 4:**
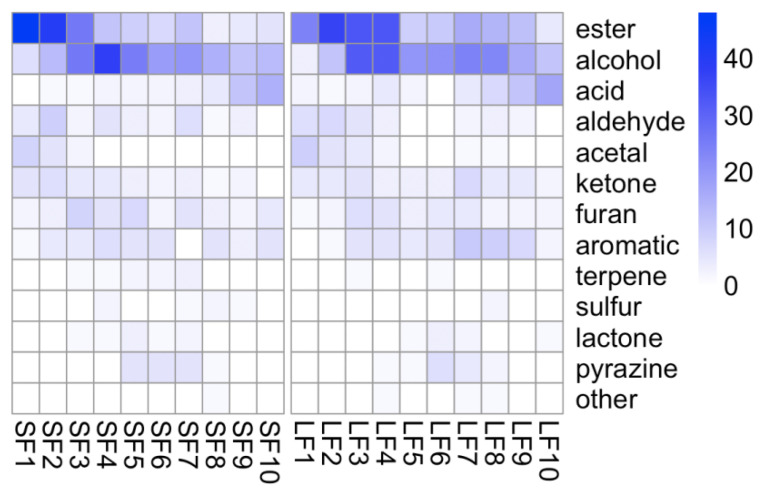
Distribution of each class of volatile compounds in LLE-based fractionation (LF) and SPE-based fractionation (SF).

The elution trend (Figure 4) of compounds extracted using SPE was quite similar to LLE, and each group of volatiles in both methods presented a similar elution trend. Similar to previous reports [23], ethyl esters and non-polar compounds were enriched from F1 to F3, alcohols were enriched in semi-polar fractions from F3 to F5, and acids were enriched in the last two fractions (Figure 3A and Figure 4). The elution trend of volatile compounds followed: esters = acetals ≤ alcohols = pyrazine = furans ≤ aromatic compounds < fatty acids.

Table 1 summarizes the number of compounds detected in each fraction from LLE and SPE extractions. More numbers of esters (F1 and F2) and alcohols (F3 to F5) were detected in SPE-based extraction, and more aromatics (F7 to F9) and acids (F10) were detected in LLE-based extraction.

### 2.4. Comprehensive Identification of Volatile Compounds in Wuliangye Baijiu

There are many brands of baijiu in China, and various techniques were developed to assess their authenticity, such as the recent advance in fluorescence spectroscopy analysis. Thus, Burns et al. [24] developed a novel fluorescence spectroscopy method to successfully distinguish different baijius. However, the essential reason for various flavor characteristics in different brands and aroma-type baijius is the difference in types and concentrations of volatiles. Therefore, it is necessary to clearly analyze volatiles in baijiu. In order to obtain the detailed chemical makeup of Wuliangye baijiu, systematic identification for each compound was carefully conducted based on critical match steps (see Section 3.5). Table S1 (Supplementary Materials) illustrates the final compounds list in Wuliangye baijiu, and a total of about 500 compounds were finally identified, although a lot of minor compounds had similarity and S/N less than 800 and 20, respectively.

#### 2.4.1. Skeleton Compounds

##### Esters

Esters, contributing to fruity and floral aromas, presented a dominant role in odor contribution in Chinese baijiu, especially the strong aroma type baijiu [2,20,25]. In this study, ester was the main class of aroma compounds in Wuliangye baijiu. A total of 122 esters were positively identified, including ethyl esters, methyl esters, nitrogen-containing ester, branched-chain methyl ester, and branched hydroxy chain (Supplementary Materials Table S1). Most of these compounds were enriched in F1 and F2 (Figure 3A). Some hydroxy branched esters, such as ethyl 2-hydroxy-4-methylpropanoate, isobutyl 2-hydroxypropanoate, ethyl 2-hydroxy-4-methylpropanoate, ethyl 2-hydroxyhexanoate, and ethyl 3-hydroxybutanoate, were identified (Supplementary Materials Table S1). Ethyl 2-hydroxy-4-methylpropanoate has been reported to contribute to blackberry aroma in wine [26], and its contribution to Wuliangye baijiu as well as other Chinese baijiu needs to be further confirmed.

##### Alcohols

A total of 89 alcohols were identified in this study, included straight-chain, methyl branched-chain, hydroxy branched-chain, and enolic alcohols (Supplementary Materials Table S1). The results agreed with the previous research that more alcohols can be identified in strong aroma type baijiu by fractionation method [2]. In this study, most of these alcohols were mainly eluted out in F4. Most alcohols can contribute to fruity, green, sweet, and alcoholic odors, and the aroma is generally weak in Chinese baijiu. Some of these alcohols could be taste-active compounds. 1-Propanol, isoamyl alcohol, 2-methyl-1-propanol, and 1-butanol have been reported to exhibit bitter and astringent taste [27]. Further research is needed to confirm their taste effect in baijiu and determine the taste contribution of other alcohols that were identified in this study in Wuliangye baijiu.

##### Acids

Fatty acids play essential roles in Wuliangye baijiu and other Chinese baijiu. They contributed to rancid, sweaty, and cheesy notes. A total of 29 fatty acids were identified in this study, and they were enriched in F9 and F10 (Supplementary Materials Table S1), including straight-chain, methyl branched, hydroxy branched, ethyl branched, and enolic acid compounds from C_2_ to C_18_. It is well known that hexanoic acid, 3-methylbutanoic acid, butanoic acid, and acetic acid gave strong aroma contribution to the overall aroma of baijiu [20]. Hydrocinnamic acid, one of the astringent compounds in red wine [28], was also identified in this study.

#### 2.4.2. Aldehydes, Ketones, and Phenolics

A total of 33 aldehydes were identified in this study (Supplementary Materials Table S1) and included straight-chain saturated/unsaturated, branched saturated/unsaturated, and hydroxy branched aldehydes. Most of the aldehydes include propanal, butanal, pentanal, hexanal, decanal, (*E,E*)-2,4-decadienal, and (*E,E*) 2,4-heptadienal, which contributed to green, grassy, and malty aromas.

A total of 58 phenolic compounds, including phenol, naphthalene, and their derivatives, were identified (Supplementary Materials Table S1). Some minor compounds, such as cresol isomers (*p*-, *o*-, and *m*-), derivates of guaiacol, methylbenzaldehyde isomers (2-methyl-, 3-methyl-), and naphthalene, were identified. Some of these compounds might present very important odor contributions to the overall aroma of baijiu due to their low sensory thresholds.

#### 2.4.3. Acetals and Furans

Acetals could be the marker for liquor aging because it is formed from the condensation of aldehydes with alcohols. As demonstrated in Figure 3C, 1,1-diethoxyethane (1) and 1,1-diethoxy-3-methylbutane (4) are the main acetals in F2. 1,1-Diethoxy-3-methylbutane was detected as one of the most important aroma compounds in Wuliangye baijiu with the FD value (which means the maximum dilution value in aroma extract dilution analysis; the aroma compounds which are still perceived at the highest dilutions are considered to be the main odor contributors.) of 4096, while 1,1-diethoxyethane was detected coeluted with ethyl acetate and detected the same FD value of 256 [20]. In-depth separation of acetals with ethyl esters and determining their flavor contribution could be important work in the future.

Furans could be another group of aroma compounds in this study. Some *γ*-lactones (e.g., butyrolactone, pentalactone, hexalactone, etc.) that contributed to sweet, coconut aromas were identified, and other furanone were also detected. In addition, 2-furanmethanol contributed to burnt sugar odor; 2-acetyl-5-methylfuran contributed to green and roast odors; and ethyl furoate contributed to balsamic odor.

#### 2.4.4. Sulfurs, Pyrazines, and Terpenes

A total of 17 sulfur-containing compounds, 14 pyrazines, and 14 terpenes were identified in this study (Supplementary Materials Table S1). Most sulfur compounds have distinctive aromas, and sometimes were considered as the off-flavor in foodstuff. Pyrazine is a group of nitrogen-containing compounds contributing to toast, nutty, and baked odors [29]. It was detected in Chinese baijiu because it could be formed in the high-temperature fermentation process of “Daqu” starter. Linalool and *β*-damascenone were important aroma compounds that contributed to floral and berry odors.

## 3. Materials and Methods

### 3.1. Sample, Solvents, Sorbents, and Standards

A typical Wuliangye baijiu sample was collected from Wuliangye Yibin Co., Ltd., Yibin city, Sichuan province, China (500 mL and the alcoholic strength of 52% by volume). The sample was stored in a dry and dark storage room.

Dichloromethane (CH_2_Cl_2_) and anhydrous sodium sulfate (Na_2_SO_4_) of ACS grade were purchased from Aladdin (Shanghai, China). Absolute ethanol and methanol with HPLC purity were purchased from J&K Technologies (Shanghai, China). Pentane with analytical grade was purchased from Xilong Chemical Co., Ltd. (Guangzhou, China) and redistilled prior to use. Hydrochloric acid (HCl) and sodium carbonate (Na_2_CO_3_) with analytical grade were purchased from Chron Chemicals (Chengdu, China). Ultrapure water was prepared using a Milli-Q purification system (Millipore, Bedford, MA, USA). LiChrolut^®^ EN (pore size of 40–120 μm) was purchased from Merck (Darmstadt, Germany). Standard SPE cartridge with no sorbent was purchased from Agilent Technologies (Palo Alto, CA, USA).

Authentic chemical standards with the GC purity listed in Table S1 were purchased from TCI (Shanghai, China), Sigma-Aldrich (Shanghai, China), Acros Technologies (Shanghai, China), Aladdin (Shanghai, China), and J&K Technologies (Shanghai, China). A mixture of *n*-alkane (C_7_ – C_30_) was purchased from Sigma-Aldrich (Shanghai, China).

### 3.2. Aroma Extraction

#### 3.2.1. LLE

An aliquot of 50 mL of baijiu was diluted to 10% ethanol (*v*/*v*) with ultrapure water, and 100 mL of CH_2_Cl_2_ was used for aroma extraction. After shaking for 30 min in a separation funnel, the organic layer was collected. The aqueous layer was extracted two more times, and all the extracts were combined and then concentrated to 50 mL under the stream of nitrogen.

#### 3.2.2. LiChrolut^®^ EN SPE

A SPE column was prepared by packing 0.5 g of LiChrolut^®^ EN resins in a 20 mL empty SPE cartridge. Before use, the column was washed sequentially with 30 mL of CH_2_Cl_2_, methanol, and 10% ethanol–water mixture. An aliquot of 50 mL of baijiu was diluted to 10% ethanol (*v*/*v*) with ultrapure water. Next, the diluted sample was percolated at 3 mL/min under the vacuum. After the sample was loaded, the SPE bed was rinsed with 30 mL of ultrapure water. The bed was then dried, and volatile compounds were finally eluted with 50 mL of CH_2_Cl_2_ for further fractionation.

### 3.3. Fractionation

Prior to the SPE fractionation, the LLE and SPE extracts were separated into an acidic fraction (AF) and a neutral/basic fraction (NBF), respectively. An aliquot of 25 mL of Na_2_CO_3_ (0.5 mol/L) was added to the extracts and mixed well. The organic layer was collected and labeled as ‘NBF’. The aqueous phase was adjusted to pH 2 with 2 mol/L HCl, then extracted twice with 50 mL aliquot of CH_2_Cl_2_, and finally, the two extracts were combined and saved as acidic fraction (F10).

The NBF was dried with anhydrous Na_2_SO_4_ and concentrated to 500 μL under a stream of nitrogen. The same SPE cartridge was used for fractionation. The concentrated NBF was carefully applied drop by drop into the SPE bed according to the procedures described previously [23]. After loading the sample, a serial of elution solvents (50 mL each) was sequentially applied for fractionation, including 100% pentane (F1), 2% CH_2_Cl_2_ in pentane (F2), 5% CH_2_Cl_2_ in pentane (F3), 10% CH_2_Cl_2_ in pentane (F4), 15% CH_2_Cl_2_ in pentane (F5), 20% CH_2_Cl_2_ in pentane (F6), 50% CH_2_Cl_2_ in pentane (F7), 100% CH_2_Cl_2_ (F8), and 5% methanol in CH_2_Cl_2_ (F9). All fractions obtained above and F10 were dried with anhydrous Na_2_SO_4_ overnight and concentrated to 500 μL under a stream of nitrogen at ambient temperature for GC×GC-TOFMS analysis.

### 3.4. Comprehensive GC×GC-TOFMS Analysis

Analysis of volatile compounds in each fraction was conducted using a GC×GC-TOFMS system including an Agilent 7890A (Agilent Technologies, Inc., Palo Alto, CA, USA) equipped with a Pegasus 4D time of flight mass spectrometer (LECO Corporation, St. Joseph, MI, USA) and Gerstel MPS 2L auto sampler (Gerstel, Mülheim, Germany). The column set consisted of a polar column (DB-WAXext, 30 m × 0.25 mm × 0.25 μm, Agilent Technologies, Inc., Palo Alto, CA, USA) as the first dimensional (^1^D) column and a DB-17SiMS (1.8 m × 0.18 mm × 0.18 μm, Agilent Technologies, Inc. Palo Alto, CA, USA) as the second dimensional (^2^D) column. A modulation period of 4.0 s was used with cryogenic trap cooled to −196 °C by liquid nitrogen.

Separation of volatile compounds in fraction was achieved using the following temperature gradient program for the ^1^D GC oven: The initial temperature of 40 °C was held for 3 min, then ramped up to 230 °C at 6 °C/min, and finally held for 10 min. The temperature program for the ^2^D GC oven was the shift of +15 °C according to the ^1^D GC oven program. The GC injector was carried out in split or splitless mode at 230 °C accordingly. Helium was used as the carrier gas at a constant flow rate of 1.0 mL/min. The temperatures of transfer line and ion source were maintained at 250 °C and 230 °C, respectively. The mass spectrometer was operated in the EI mode at 70 eV, and the voltage was set to −1500 V. Ions in the *m*/*z* range of 35–350 were acquired with a data acquisition rate of 100 spectra/s. Total ion chromatogram (TIC) contour plots were processed using the automated data software ChromaTOF (version 4.32, LECO, St. Joseph, MI, USA).

### 3.5. Identification of Volatile Compounds

An integrated method was developed and applied for the identification of volatile compounds. The peak finding process on the basis of the automated deconvolution method was tentatively identified with the following criteria: (a) comparison of their mass spectra to a reference database (MS, NIST 17 database) in ChromaToF chemstation. A mass spectral match factor—all of the tentatively identified compounds showed similarity matches more than 800—was set to decide whether a peak was correctly identified or not. (b) A comparison of their retention time and mass spectra with authentic chemical standards (in Supplementary Materials Table S1 represented as Std). (c) A comparison of the linear retention indices that calculated RI with the values reported in the literature (RIL) for the DB-Wax (or equivalent column) and the authentic chemical standards in the same detection program (Std). A mixture of C_7_ – C_30_ *n*-alkanes was used for the calculation of linear RI for each compound [30].

### 3.6. Data Analysis

In order to well understand the distribution trend of each kind of volatile compound as well as the single compound, the peak area ratio with the value greater than 0.2% in each contour total ion chromatogram was selected. Venny 2.1 program [31] was conducted to summarized the distinction of volatile compounds between LLE- and SPE-based fractionation. R program (v4.0.3) [32] was employed to summarize the distribution of the main class of volatile compounds in each fraction.

## 4. Conclusions

An extraction and comprehensive fractionation technique based on LiChrolut^®^ EN resin was achieved for the fractionation of volatile compounds in Wuliangye baijiu. This technique can reduce the interference of volatile compounds on each other and facilitate other volatile compound identification and analysis. Furthermore, the technique is combined with GC×GC-TOFMS, which allows for the separation of a large number of compounds in a single chromatographic run due to the added selectivity of the second column and inherently high peak capacity, so that more trace compounds can be identified.

Volatile compounds with different polarities were separated into different fractions. The fractionation simplified complex volatile composition in Wuliangye baijiu based on their functional group, and comprehensive GC×GC-TOFMS analysis on each fraction facilitated the reliable identification. This technique enables the identification of about 500 volatile compounds, including some minor compounds such as hydroxy-branched esters, furans, and phenolic compounds in Wuliangye baijiu. This systematic analytical approach can be used for the characterization of all other baijius.

## 5. Patents

This section is not mandatory but may be added if there are patents resulting from the work reported in this manuscript.

## Figures and Tables

**Table 1:** Number of volatile compounds identified in each fraction.

	Ester		Alcohol		Acid		Aldehyde		Acetal		Ketone		Furan		Phenolic		Terpene		Sulfur		Lactone		Pyrazine		Other	
	SPE	LLE	SPE	LLE	SPE	LLE	SPE	LLE	SPE	LLE	SPE	LLE	SPE	LLE	SPE	LLE	SPE	LLE	SPE	LLE	SPE	LLE	SPE	LLE	SPE	LLE
**F1**	48	24	6	3	0	2	4	6	8	9	5	4	2	1	1	0	0	0	0	0	0	0	0	0	0	0
**F2**	40	37	13	11	1	1	9	7	5	5	6	4	3	2	4	1	0	0	0	0	0	0	0	0	0	0
**F3**	26	33	26	32	1	2	2	5	2	4	4	5	8	6	4	5	1	1	0	0	1	0	0	0	0	0
**F4**	11	33	38	32	2	4	5	3	0	2	4	3	5	5	6	5	1	0	2	0	1	0	0	1	0	1
**F5**	9	9	25	20	2	2	3	0	0	0	3	3	7	3	5	4	2	0	0	0	3	1	5	1	0	0
**F6**	7	10	19	21	2	0	2	0	0	0	2	3	2	4	5	5	2	1	0	0	1	3	5	6	0	0
**F7**	11	16	20	24	3	4	6	2	0	1	3	7	5	4	0	10	3	0	1	0	2	2	5	4	0	1
**F8**	3	14	15	23	4	7	1	3	0	1	1	4	3	2	5	9	0	0	2	2	0	0	1	2	1	1
**F9**	4	12	11	16	11	11	3	2	0	0	2	4	2	2	3	7	0	0	1	0	0	0	0	0	0	0
**F10**	5	4	13	11	15	17	0	0	0	0	0	2	4	2	5	2	0	0	0	0	0	1	0	0	0	0

## Data Availability

The data presented in this study are available on request from the corresponding author.

## Supplementary Materials

The following are available online, Table S1: Volatile compounds in Wuliangye baijiu using comprehensive SPE fractionation coupled with GC×GC-TOFMS. Figure S1: MS spectra of (A) ethyl hydrogen malonate; (B) 1,1-diethoxy-3-methyl-butane; (C) hexyl acetate; (D) benzeneacetaldehyde.

## Sample Availability

Samples of the compounds are available from the authors.
