# Supplementary material for: Volatile Analysis of Wuliangye Baijiu by LiChrolut EN SPE Fractionation Coupled with Comprehensive GC×GC-TOFMS
Source: Molecules. 2022 Feb 15;27(4):1318. doi: 10.3390/molecules27041318 (PMC8878284; doi:10.3390/molecules27041318)
Supplement: Supplementary file 1 [file molecules-27-01318-s001.zip › molecules-1567954-supplementary.pdf]

Supplementary Materials

Volatile analysis of Wuliangye baijiu by LiChrolut EN SPE fractionation coupled with comprehensive GC×GC-TOF-Mass spectrometry

Jia Zheng [1,*], Zhanglan He [1], Kangzhuo Yang [1], Zhipeng Liu [1], Dong Zhao [1] and Michael C. Qian [2,*]

[1] Flavor Science Innovation Center, Technology Research Center, Wuliangye Yibin Co., Ltd., 150# Minjiang West Road, Cuiping District, Yibin 644000, China
[2] Department of Food Science and Technology, Oregon State University, Corvallis, OR, USA 97331
* Correspondence: zhengwanqi86@163.com (J.Z.); michael.qian@oregonstate.edu (M.Q.)

Table S1. Volatile compounds in Wuliangye baijiu using comprehensive SPE fractionation coupled with GC×GC-TOFMS.

Number	Compound	RIwax[a]	RIL[b]	^{2}D RT (s)	Identification[c]
Esters					
1	Methyl acetate	863	864	1.39	MS,Std,RI
2	Ethyl acetate	895	890	1.52	MS,Std,RI
3	Ethyl propanoate	951	956	1.68	MS,Std,RI
4	Ethyl isobutyrate	960	961	1.85	MS,Std,RI
5	*n*-Propyl acetate	962	969	1.62	MS,Std,RI
6	Methyl butanoate	974	976	1.64	MS,Std,RI
7	*sec*-Butyl acetate	978	982	1.73	MS,Std,RI
8	Ethyl 2-propenoate	981	980	1.58	MS,Std,RI
9	Methyl 2-methylbutanoate	1002	1009	1.73	MS,Std,RI
10	Isobutyl acetate	1005	1000	1.72	MS,Std,RI
11	Butyl formate	1014	1033	1.56	MS,Std,RI
12	Ethyl butanoate	1026	1026	1.86	MS,Std,RI
13	Propyl propanoate	1047	1045	1.78	MS,Std,RI
14	Ethyl 2-oxobutanoate	1056		1.97	MS,Tent
15	Ethyl 2-methyl- butanoate	1047	1065	1.90	MS,Std,RI
16	Methyl 3-methyl-2-oxopentanoate	1051		2.94	MS,Tent
17	Ethyl isopentanoate	1068	1064	1.87	MS,Std,RI
18	Butyl acetate	1074	1075	1.74	MS,Std,RI
19	Methyl valerate	1101	1109	1.76	MS,Std,RI
20	2-Methylbutyl acetate	1128		1.80	MS,Std
21	3-Methylbutyl acetate	1134	1140	1.84	MS,Std,RI
22	2-Pentanol, acetate	1134	1074	1.78	MS,RI,Tent
23	Propyl butanoate	1134	1139	1.93	MS,Std,RI
24	Ethyl pentanoate	1142	1134	1.93	MS,Std,RI
25	Butyl propanoate	1148		1.90	MS,Std
26	Ethyl 2-methylbutanoate	1150	1141	2.11	MS,Std,RI
27	Isobutyl butanoate	1162	1161	1.98	MS,Std,RI
28	Ethyl (Z)-2-butenoate	1172		1.67	MS,Std
29	Pentyl acetate	1179	1175	1.79	MS,Std,RI
30	2-Propenyl butanoate	1181	1185	1.72	MS,Std,RI
31	Methyl hexanoate	1193	1177	1.85	MS,Std,RI
32	Ethyl 4-methylpentanoate	1197	1197	1.95	MS,Std,RI
33	Butyl butanoate	1222	1215	2.00	MS,Std,RI

34	Ethyl hexanoate	1241	1251	2.00	MS,Std,RI
35	Isoamyl butanoate	1275	1255	2.06	MS,Std,RI
36	Hexyl acetate	1282	1287	1.87	MS,Std,RI
37	2-Methylpropyl pantanoate	1283	1252	2.01	MS,Std,RI
38	Butyl pentanoate	1283	1296	2.02	MS,Std,RI
39	Ethyl ethoxyacetate	1305		1.65	MS,Std
40	Ethyl 5-methylhexanoate	1308		2.01	MS,Tent
41	Methyl heptanoate	1310	1302	1.91	MS,Std,RI
42	Hex-4-enoic acid, ethyl ester (other isomer)	1317		1.83	MS,Tent
43	Methyl 2-hydroxy-2-methylbutanoate	1320	1281	1.54	MS,Std,RI
44	Ethyl (*E*)-3-hexenoate	1322	1292	1.84	MS,RI,Tent
45	2-Pentyl pentanoate	1325		2.14	MS,Std
46	Methyl lactate	1331	1314	1.36	MS,Std,RI
47	Propyl hexanoate	1332	1321	2.05	MS,Std,RI
48	(*S*)-Isopropyl lactate	1339		1.44	MS,Tent
49	Isobutyl hexanoate	1339	1356	2.20	MS,Std,RI
50	Ethyl heptanoate	1346	1349	2.01	MS,Std,RI
51	Ethyl 3-ethoxypropanoate	1351	1320	1.74	MS,RI,Tent
52	Hexyl propanoate	1358	1349	2.02	MS,Std,RI
53	Hexyl butanoate	1361	1388	2.14	MS,Std,RI
54	Ethyl lactate	1363	1371	1.40	MS,Std,RI
55	Ethyl methoxyacetate	1367		1.41	MS,Std
56	Isopentyl pentanoate	1377	1346	2.16	MS,Std,RI
57	2-Propenyl hexanoate	1384	1371	1.86	MS,RI,Tent
58	Heptyl acetate	1389	1364	1.95	MS,Std,RI
59	Methyl octanoate	1404	1399	1.95	MS,Std,RI
60	3-Pentyl hexanoate	1417		2.25	MS,Tent
61	Ethyl 2-hydroxybutanoate	1424	1400	1.45	MS,RI,Tent
62	Butyl hexanoate	1427	1416	2.11	MS,Std,RI
63	Ethyl 3-hydroxy-3-methylbutanoate	1429	1407	1.53	MS,RI,Tent
64	Isobutyl (*R*)-(+)-lactate	1434	1455	1.50	MS,RI,Tent
65	1,1-Ethanediol, diacetate	1436		1.55	MS,Tent
66	Ethyl 2,2-diethoxypropionate	1439		1.87	MS,Tent
67	Ethyl 2-hydroxy-3-methylbutanoate	1444	1426	1.54	MS,Std,RI
68	Ethyl octanoate	1454	1440	2.13	MS,Std,RI
69	Ethyl 2-(1-ethoxyethoxy)propanoate	1457	1442	1.82	MS,RI,Tent
70	Hexyl 3-methylbutanoate	1460	1425	2.21	MS,RI,Tent

71	Isopentyl hexanoate	1472	1464	2.19	MS,Std,RI
72	2-Pentyl methoxyacetate	1513		1.55	MS,Tent
73	2-Butenyl hexanoate	1513		1.97	MS,Tent
74	Pentyl hexanoate	1527	1525	2.18	MS,Std,RI
75	Ethyl 2-hydroxy-2-methylpropanoate	1531		1.31	MS,Tent
76	Ethyl 3,3-diethoxypropionate	1532		1.81	MS,Tent
77	Propyl octanoate	1532	1539	2.15	MS,Std,RI
78	Ethyl 3-hydroxybutanoate	1534	1546	1.45	MS,Std,RI
79	Butyl lactate	1534	1520	1.51	MS,Std,RI
80	Methyl 2-hydroxy-4-methylpentanoate	1540	1522	1.47	MS,Std,RI
81	Ethyl nonanoate	1548	1541	2.13	MS,Std,RI
82	Ethyl DL-leucate	1558	1547	1.59	MS,Std,RI
83	Isobutyl hexanoate	1564	1551	2.20	MS,Std,RI
84	Isopentyl heptanoate	1569	1548	2.22	MS,Std,RI
85	1,2-Propanediol, 1-acetate	1582	1579	1.37	MS,RI,Tent
86	Pentyl methoxyacetate	1582		1.55	MS,Tent
87	3-Methylbutyl methoxyacetate	1582	1570	1.56	MS,RI,Tent
88	Isoamyl lactate	1585	1572	1.51	MS,Std,RI
89	Methyl levulate	1588	1560	1.51	MS,Std,RI
90	Diethyl malonate	1590	1572	1.59	MS,Std,RI
91	Butyl butryllactate	1591		1.77	MS,Tent
92	2-Heptyl hexanoate	1596	1591	2.28	MS,RI,Tent
93	Ethyl 2-hydroxyhexanoate	1520	1544	1.54	MS,RI,Tent
94	Hexyl hexanoate	1622	1612	2.19	MS,Std,RI
95	1,2-Propanediol, 2-acetate	1624	1621	1.36	MS,RI,Tent
96	Ethyl levulinate	1627	1614	1.61	MS,Std,RI
97	Pentyl formate	1633		1.58	MS,Tent
98	Ethyl decanoate	1650	1659	2.14	MS,Std,RI
99	Diethyl methylsuccinate	1655		1.72	MS,Std,RI
100	Isoamyl octanoate	1670	1668	2.23	MS,Std,RI
101	Diethyl succinate	1690	1690	1.66	MS,Std,RI
102	Ethyl carbamate	1692		1.30	MS,Std
103	Ethyl 3-hydroxyhexanoate	1695	1675	1.53	MS,Std,RI
104	vinyl hexanoate	1719		1.72	MS,Std
105	Hexyl methoxyacetate	1737		1.56	MS,Tent
106	Ethyl 4-acetylbutanoate	1746		1.61	MS,Std
107	Ethyl undecanoate	1753	1732	2.13	MS,Std,RI

108	Ethyl dodecanoate	1854	1865	2.21	MS,Std,RI
109	Methyl 9,12,15-octadecatrienoate	1862			MS
110	Ethyl tetradecanoate	2060	2057	2.38	MS,Std,RI
111	Diethyl DL-Malate	2063	2053	1.44	MS,Std,RI
112	Ethyl pentadecanoate	2113	2135	2.33	MS,Std,RI
113	Ethyl 9-oxononanoate	2116	2160	1.73	MS,RI,Tent
114	Methyl hippurate	2127		1.63	MS,Tent
115	Ethyl hexadecanoate	2265	2246	2.33	MS,Std,RI
116	Ethyl 9-hexadecenoate	2296	2267	2.26	MS,Std,RI
117	Ethyl hydrogen succinate	2401	2368	1.32	MS,Std,RI
118	Methyl 15-methylhexadecanoate	2445		2.66	MS,Tent
119	Ethyl 9-octadecenoate	2470	2469	2.94	MS,RI,Tent
120	Ethyl E-11-hexadecenoate	2514		3.15	MS,Tent
121	Ethyl oleate	2514	2516	3.18	MS,Std,RI
122	Ethyl linoleate	2555	2532	3.25	MS,Std,RI
Alcohols					
1	4-methyl-2-propylpentan-1-ol	928		2.67	MS,Tent
2	3-Buten-2-ol	935		2.50	MS,Std
3	1-Ethoxy-2-propanol	974		1.80	MS,Std
4	2-Butanol	1020	1028	1.49	MS,Std,RI
5	1-Propanol	1026	1034	1.43	MS,Std,RI
6	3-Methyl-2-butanol	1026	1089	1.47	MS,Std,RI
7	Isoamyl alcohol	1233	1227	2.46	MS,Std,RI
8	2-Methyl-3-buten-2-ol	1035	1028	1.37	MS,RI,Tent
9	Isobutanol	1095	1098	1.43	MS,Std,RI
10	2,3-Dimethyl-butan-2-ol	1095	1082	1.52	MS,Std,RI
11	3,3-Dimethylbutane-2-ol	1095		1.57	MS,Tent
12	2-Methyl-2-pentanol	1105	1101	1.46	MS,Std,RI
13	3-Pentanol	1109	1110	1.43	MS,Std,RI
14	2-Methyl-2-butenal	1109	1093	1.60	MS,Std,RI
15	3-Methyl-3-pentanol	1119	1080	1.48	MS,Std,RI
16	(*S*)-(+)-2-Pentanol	1123		1.44	MS,Std
17	(*R*)-(-)-2-Pentanol	1123		1.41	MS,Std
18	2-Pentanol	1125	1116	1.52	MS,Std,RI
19	1-Methoxy-2-propanol	1133	1135	1.38	MS,Std,RI
20	1-Butanol	1142	1165	1.39	MS,Std,RI
21	2,3-Dimethylbut-3-en-2-ol	1150		1.48	MS,Tent
22	2-Methyl-3-pentanol	1158	1167	1.46	MS,RI,Tent

23	(*R*)-2-Hexanol	1164		1.43	MS,Tent
24	4-Methyl-2-pentanol	1166	1168	1.44	MS,RI,Tent
25	3-Penten-2-ol	1168	1183	1.38	MS,RI,Tent
26	1,5-Hexadien-3-ol	1176		1.34	MS,Tent
27	3-Hexanol	1195	1190	1.49	MS,Std,RI
28	4-Amino-1-butanol	1197		1.59	MS,Tent
29	2-Methylbutanol	1201	1206	1.37	MS,Std,RI
30	3-Methyl-2-butenal	1207	1206	1.54	MS,Std,RI
31	1-Pentanol	1207	1217	1.63	MS,Std,RI
32	2-Methyl-2-propen-1-ol	1217		1.33	MS,Tent
33	(*S*)-2-Hexanol	1217		1.45	MS,Tent
34	(*E*)-2-Buten-1-ol	1217	1215	1.32	MS,Std,RI
35	2-Buten-1-ol	1217	1193	1.36	MS,Std,RI
36	3-Methyl-2-buten-1-ol	1217	1277	1.61	MS,RI,Tent
37	(*Z*)-2-Buten-1-ol	1246		1.33	MS,Tent
38	3-Methyl-3-buten-1-ol	1256	1254	1.37	MS,Std,RI
39	(*E*)-1,3-Butadien-1-ol	1280		1.68	MS,Tent
40	(+-)-5-Methyl-2-hexanol	1285		1.47	MS,Tent
41	4-Heptanol	1290	1288	1.51	MS,Std,RI
42	(*E*)-3-Hexen-1-ol	1298	1326	1.40	MS,Std,RI
43	1-(2-Methoxyethoxy)-2-methyl-2-propanol	1303		1.41	MS,Std
44	2-Methyl-1-pentanol	1310	1337	1.42	MS,Std,RI
45	4-Penten-1-ol	1312	1289	1.36	MS,Std,RI
46	Cyclopentanol	1312	1300	1.41	MS,Std,RI
47	Prenol	1331	1320	1.39	MS,Std,RI
48	4-Methylpentanol	1322	1316	1.41	MS,Std,RI
49	(*S*)-2-Heptanol	1327		1.51	MS,Tent
50	(*Z*)-2-Penten-1-ol	1331	1332	1.37	MS,Std,RI
51	2-Methyl-2-buten-1-ol	1331	1324	1.38	MS,Std,RI
52	2-Heptanol	1332	1332	1.52	MS,Std,RI
53	3-Methylpentanol	1336	1318	1.41	MS,Std,RI
54	2-Isopropyl-5-methyl-1-heptanol	1351		2.68	MS,Tent
55	(*S*)-(+)-1,2-Propanediol	1355		1.49	MS,Std
56	1-Hexanol	1360	1360	1.44	MS,Std,RI
57	Triethylene glycol	1365		1.44	MS,Std
58	6-Methyl-2-heptanol	1382	1381	1.54	MS,Std,RI
59	5-Methyl-2-heptanol	1384	1394	1.53	MS,Std,RI

60	3-Ethoxypropanol	1389	1371	1.40	MS,Std,RI
61	4-Octanol	1389	1376	1.57	MS,Std,RI
62	(*Z*)-3-Hexen-1-ol	1398	1386	1.41	MS,Std,RI
63	3-Octanol	1401	1396	1.57	MS,Std,RI
64	(*E*)-2-Hexen-1-ol	1416	1394	1.41	MS,Std,RI
65	(*Z*)-4-Hexen-1-ol	1419	1422	1.40	MS,Std,RI
66	(*E*)-4-Hexen-1-ol	1421	1413	1.41	MS,Std,RI
67	(*R*)-2-Octanol	1424		1.54	MS,Tent
68	Ethanol, 2-butoxy-	1434	1435	1.47	MS,RI,Tent
69	3,7-Dimethyl-3-octanol	1436	1414	1.69	MS,RI,Tent
70	4-Methylhexanol	1439	1414	1.49	MS,Std,RI
71	1-Heptanol	1462	1462	1.50	MS,Std,RI
72	2,2-Diethoxyethanol	1467		1.45	MS,Std
73	6-Methyl-5-Hepten-2-ol	1472	1451	1.50	MS,Std,RI
74	2-Ethyl-1-hexanol	1497	1484	1.53	MS,Std,RI
75	4-Hepten-1-ol	1502	1502	1.45	MS,RI,Tent
76	(*S*)-3-Ethyl-4-methylpentanol	1518	1510	1.51	MS,RI,Tent
77	11-Methyldodecanol	1519		2.93	MS,Tent
78	(*E*)-2-Hepten-1-ol	1521	1536	1.44	MS,Std,RI
79	2-Nonanol	1524	1530	1.60	MS,Std,RI
80	1-Octanol	1564	1561	1.51	MS,Std,RI
81	Propylene Glycol	1598	1600	1.35	MS,Std,RI
82	3,3-Diethoxy-1-propanol	1618	1611	1.50	MS,Std,RI
83	1-Nonanol	1667	1666	1.54	MS,Std,RI
84	1-Nonen-4-ol	1713		1.56	MS,Std
85	(*S*)-2-Octanol	1725		1.65	MS,Std
86	2,5-dimethyl-2,5-hexanediol	1770		1.39	MS,Std
87	(*S*)-(-)-1,2,4-Butanetriol	1892		2.22	MS,Tent
88	1,4-Butanediol	1944	1911	1.28	MS,Std,RI
89	2,6-dimethyl-3,7-octadiene-2,6-diol	1951	1957	1.39	MS,RI,Tent
Aldehydes					
1	Propanal	835	823	1.38	MS,Std,RI
2	Isobutanal	858	834	1.46	MS,Std,RI
3	Butanal	904	905	1.48	MS,Std,RI
4	2-Methylbutanal	930	907	1.73	MS,Std,RI
5	Isopentanal	932	932	1.69	MS,Std,RI
6	Pentanal	1017	1006	1.56	MS,Std,RI
7	(*E*)-2-Butenal	1044	1050	1.49	MS,Std,RI

8	2-Butenal	1044	1038	1.49	MS,Std,RI
9	Acetaldehyde, methoxy-	1095		1.55	MS,Tent
10	Hexanal	1098	1101	1.77	MS,Std,RI
11	2-Methyl-2-butenal	1109	1088	1.59	MS,Std,RI
12	3-Methyl-2-butenal	1207	1199	1.56	MS,Std,RI
13	Heptanal	1207	1202	1.79	MS,Std,RI
14	2-Hexenal	1246	1248	1.63	MS,Std,RI
15	Octanal	1313	1311	1.85	MS,Std,RI
16	2-Ethyl-4-pentenal	1344		1.72	MS,Tent
17	(*E*)-2-Heptenal	1348	1321	1.71	MS,Std,RI
18	(*Z*)-2-Heptenal	1353	1319	1.71	MS,Std,RI
19	Nonanal	1411	1406	1.93	MS,Std,RI
20	(*E*)-2-Octenal	1454	1427	1.77	MS,Std,RI
21	3-Hydroxybutanal	1499		1.30	MS,Std
22	(*E,E*)-2,4-Heptadienal	1516	1522	1.62	MS,Std,RI
23	Decanal	1516	1498	1.94	MS,Std,RI
24	2-Methylhexanal	1532		2.06	MS,Std
25	(*E*)-2-Nonenal	1556	1553	1.79	MS,Std,RI
26	2-Methylpentanal	1613		1.94	MS,Tent
27	(*Z*)-2-Decenal	1664	1644	1.80	MS,Std,RI
28	2,4-Nonadienal	1725	1710	1.67	MS,Std,RI
29	(*E*)-2-Undecenal	1773	1753	1.83	MS,Std,RI
30	(*E,E*)-2,4-Decadienal	1785	1795	1.71	MS,Std,RI
31	2-Phenylpropenal	1832		1.59	MS,Tent
32	2,4-Decadienal	1835	1824	1.71	MS,Std,RI
33	4-Hydroxyhexenal	1968		1.47	MS,Tent
Phenolics					
1	Indane	1396	1377	1.93	MS,Std,RI
2	Benzeneacetaldehyde, à,2,5-trimethyl-	1487		2.17	MS,Tent
3	Benzaldehyde	1553	1549	1.57	MS,Std,RI
4	1,2,3,4-Tetrahydronaphthalene	1561	1565	2.03	MS,Std,RI
5	1,2,3,4-Tetrahydro-2-methylnaphthalene	1593	1685	2.05	
6	1,2,3,4-Tetrahydro-1-methylnaphthalene	1607		2.11	MS,Tent
7	Benzoyl isothiocyanate	1653		1.64	MS,Tent
8	2-Methylbenzaldehyde	1655	1622	1.62	MS,Std,RI
9	3-Methylbenzaldehyde	1655	1628	1.62	MS,Std,RI
10	4-Methylbenzaldehyde	1655	1655	1.64	MS,Std,RI

11	1,2,3,4-Tetrahydro-5-methylnaphthalene	1667		2.05	MS,Tent
12	Benzeneacetaldehyde	1672	1674	1.56	MS,Std,RI
13	Acetophenone	1684	1694	1.59	MS,Std,RI
14	Ethyl benzoate	1693	1673	1.71	MS,Std,RI
15	4-Hydroxybenzaldehyde	1693		1.67	MS,Tent
16	1,2,3,4-Tetrahydro-1,4-dimethylnaphthalene	1696		2.11	MS,Std
17	2-Hydroxybenzaldehyde	1713	1689	1.55	MS,Std,RI
18	1,2,3,4-Tetrahydro-6-methylnaphthalene	1728		2.04	MS,Std
19	1-Phenyl-2-propanone	1758	1710	1.61	MS,Std,RI
20	Benzaldehyde, 3-hydroxy-4-methoxy-	1768		2.92	MS,Std
21	1-Hydroxycumene	1776	1776	1.47	MS,Std,RI
22	3-Hydroxy-3-phenylbutan-2-one	1779		1.47	MS,Tent
23	Naphthalene	1779	1791	1.74	MS,Std,RI
24	1,2-dihydro-1,1,6-trimethylnaphthalene	1780	1748	2.02	MS,RI,tent
25	Furfural isovalerate	1785		1.70	MS,Std
26	Ethyl benzeneacetate	1810	1785	1.72	MS,Std,RI
27	Vinyl benzoate	1829		1.73	MS,Tent
28	1-Phenethyl alcohol	1835	1820	1.41	MS,RI,tent
29	2-Phenylethyl acetate	1841	1828	1.68	MS,Std,RI
30	Guaiacol	1889	1894	1.42	MS,Std,RI
31	1-Methylnaphthalene	1892	1878	1.81	MS,Std,RI
32	Propyl phenylacetate	1892	1848	1.79	MS,Std,RI
33	2-Methylnaphthalene	1895	1877	1.82	MS,Std,RI
34	Benzyl alcohol	1901	1895	1.36	MS,Std,RI
35	Ethyl hydrocinnamate	1911	1892	1.78	MS,Std,RI
36	Phenylethyl Alcohol	1938	1938	1.44	MS,Std,RI
37	α-ethylidenebenzeneacetaldehyde	1964	1933	1.67	MS,RI,tent
38	Quinoline	1981	1942	1.65	MS,Std,RI
39	Butyl phenylacetate	1988	1970	1.83	MS,Std,RI
40	α-Phenylethyl butyrate	1991	1978	1.86	MS,Std,RI
41	1,7-Dimethylnaphthalene	2004	2000	1.86	MS,Std,RI
42	*o*-Cresol	2025	2030	1.31	MS,Std,RI
43	Isopentyl phenylacetate	2029	2016	1.87	MS,RI,tent
44	Phenol	2032	2023	1.31	MS,Std,RI
45	1,3-Dimethylnaphthalene	2046	2015	1.85	MS,Std,RI
46	3-Phenylpropanol	2074	2048	1.47	MS,RI,tent
47	*p*-Cresol	2108	2096	1.31	MS,Std,RI

48	*m*-Cresol	2109	2012	1.33	MS,Std,RI
49	2-Hydroxy-iso-butyrophenone	2112		1.51	MS,Tent
50	2-Phenoxyethanol	2174	2142	1.43	MS,Std,RI
51	2-Phenylethyl hexanoate	2200	2160	1.93	MS,Std,RI
52	2-Ethylphenol	2203	2050	1.35	MS,Std,RI
53	4-Ethylphenol	2203	2183	1.34	MS,Std,RI
54	3-Ethylphenol	2211	2210	1.34	MS,Std,RI
55	4-Vinylguaiacol	2226	2203	1.46	MS,Std,RI
56	2,6-Dimethoxyphenol	2295	2269	1.51	MS,Std,RI
57	Benzoic acid	2474	2436	1.32	MS,Std,RI
58	3-Hydroxy-4-methoxybenzyl alcohol	2516		1.69	MS,Std
Furans					
1	Dihydro-2-methyl-3(2H)-furanone	1298	1260	1.50	MS,Std,RI
2	3-Furaldehyde	1451	1455	1.40	MS,Std,RI
3	2-Furaldehyde diethyl acetal	1480	1442	1.76	MS,RI,Tent
4	Furfural	1487	1494	1.45	MS,Std,RI
5	2-Acetyl-5-methylfuran	1516	1593	1.56	MS,RI,Tent
6	2-Acetyfuran	1532	1538	1.48	MS,Std,RI
7	Tetrahydro-2,2,4,4-tetramethyl-3-Furanol	1550		1.43	MS,Tent
8	2-Furanmethanol, acetate	1553	1541	1.54	MS,Std,RI
9	5-Methyl-2-furfural	1598	1608	1.50	MS,Std,RI
10	Furyl ethyl ketone	1601	1569	1.54	MS,Std,RI
11	Dihydro-3,5-dimethyl-2(3H)-furanone	1607		1.59	MS,Tent
12	Furfuryl propanoate	1615	1620	1.60	MS,Std,RI
13	2,2'-Methylenebisfuran	1633	1615	1.59	MS,RI,Tent
14	5,5-Dimethyl-2(5H)-furanone	1638	1590	1.49	MS,RI,Tent
15	Ethyl 2-furoate	1644	1628	1.55	MS,Std,RI
16	γ-Pentalactone	1644	1648	1.51	MS,Std,RI
17	3-Methylbutyrolactone	1650		1.51	MS,Std
18	γ-Butyrolactone	1664	1666	1.48	MS,Std,RI
19	Benzeneacetaldehyde	1673	1680	1.60	MS,Std,RI
20	2-Furanmethanol	1675	1669	1.31	MS,Std,RI
21	Furfuryl butanoate	1687	1649	1.65	MS,Std,RI
22	Lavender lactone	1701	1684	1.55	MS,Std,RI
23	5-Methyl-2(5H)-furanone	1710	1664	1.43	MS,RI,Tent
24	γ-Hexalactone	1737	1724	1.57	MS,Std,RI
25	3-Methyl-2(5H)-furanone	1752	1726	1.46	MS,Std,RI

26	5-Ethoxydihydro-2(3H)-furanone	1755	1794	1.53	MS,RI,Tent
27	Dihydro-3,3-dimethyl-2(3H)-furanone	1764		1.56	MS,Std
28	3,4-Dimethyl-2,5-furandione	1764	1764	1.50	MS,Std,RI
29	5-Methoxy-2,5-dihydrofuran-2-one	1770		1.40	MS,Tent
30	Furfuryl pentanoate	1785	1753	1.70	MS,Std,RI
31	2(5H)-Furanone	1791	1767	1.39	MS,Std,RI
32	γ-Heptalactone	1838	1784	1.58	MS,Std,RI
33	Furan acrolein	1885	1851	1.46	MS,Std,RI
34	3-Phenylfuran	1886	1880	1.64	MS,Std,RI
35	Furfuryl hexanoate	1886	1857	1.76	MS,Std,RI
36	γ-Octalactone	1951	1916	1.61	MS,Std,RI
37	Furfuryl heptanoate	1988	1950	1.80	MS,Std,RI
38	Pantolactone	2066	2077	1.32	MS,Std,RI
39	γ-Nonalactone	2067	2028	1.65	MS,Std,RI
40	2-Furancarboxylic acid	2462		1.32	MS,Std
41	5-Hydroxymethyldihydrofuran-2-one	2550		1.42	MS,Std
Ketones					
1	Acetone	854	823	1.37	MS,Std,RI
2	2-Butanone	920	909	1.49	MS,Std,RI
3	3-Methyl-2-butanone	934	927	1.64	MS,Std,RI
4	Methyl vinyl ketone	957	932	1.43	MS,Std,RI
5	2-Pentanone	969	985	1.70	MS,Std,RI
6	2,3-Butanedione	973	979	1.40	MS,Std,RI
7	3-Pentanone	976	971	1.62	MS,RI,Tent
8	3-Methyl-3-buten-2-one	987	987	1.54	MS,RI,Tent
9	2-Methyl-3-pentanone	988	1000	1.72	MS,RI,Tent
10	Methyl isobutyl ketone	997	1006	1.67	MS,Std,RI
11	3-Methyl-2-pentanone	1011	1011	1.69	MS,Std,RI
12	2,2-Dimethyl-3-hexanone	1027		2.53	MS,RI,Tent
13	2-Hexanone	1092	1088	1.74	MS,Std,RI
14	(*E*)-3-Penten-2-one	1138	1108	1.53	MS,RI,Tent
15	5-Methyl-2-hexanone	1168	1146	1.68	MS,RI,Tent
16	2-Heptanone	1189	1174	1.79	MS,Std,RI
17	Cyclopentanone	1195	1186	1.65	MS,Std,RI
18	4-Ethoxy-2-butanone	1285		1.60	MS,RI,Tent
19	3-Octanone	1285	1272	1.84	MS,Std,RI
20	Acetoin	1305	1287	1.36	MS,Std,RI
21	2-Octanone	1310	1297	1.85	MS,Std,RI

22	Cyclohexanone	1317	1314	1.73	MS,Std,RI
23	1-Hydroxy-2-propanone	1322	1320	1.31	MS,Std,RI
24	4-Nonanone	1346	1357	1.97	MS,RI,Tent
25	6-Methyl-5-hepten-2-one	1360	1341	1.76	MS,Std,RI
26	3-Ethylcyclopentanone	1365		1.84	MS,RI,Tent
27	2-Cyclopenten-1-one	1382	1316	1.54	MS,Std,RI
28	3-Nonanone	1384	1384	1.90	MS,Std,RI
29	4-Hydroxy-4-methyl-2-pentanone	1389	1390	1.42	MS,RI,Tent
30	2-Nonanone	1406	1390	1.91	MS,Std,RI
31	3-Octen-2-one	1431	1414	1.76	MS,Std,RI
32	2,3-dimethyl-2-cyclopenten-1-one	1472	1530	1.74	MS,RI,Tent
33	4-Hydroxy-2-pentanone	1479		1.35	MS,RI,Tent
34	2-Octen-4-one	1497		1.85	MS,RI,Tent
35	2,5-Hexanedione	1526	1515	1.52	MS,RI,Tent
36	3-Nonen-2-one	1532	1508	1.78	MS,Std,RI
37	4-Undecanone	1548	1540	2.08	MS,Std,RI
38	3-Undecanone	1580	1586	2.04	MS,Std,RI
39	(*E,E*)-3,5-Octadien-2-one	1590	1590	1.65	MS,RI,Tent
40	5,5-diethoxy-2-pentanone	1591	1563	1.76	MS,RI,Tent
41	2-Undecanone	1616	1599	1.94	MS,Std,RI
42	1-phenylpropanone	1755	1734	1.66	MS,RI,Tent
43	3-Hydroxy-3-phenylbutan-2-one	1776		1.47	MS,RI,Tent
44	3-Tridecanone	1789	1755	2.07	MS,RI,Tent
45	1,6-Dioxacyclododecane-7,12-dione	2368		1.86	MS,RI,Tent
Sulfur, Pyrazines, and Terpenes					
1	Sulfur dioxide	886	882	1.25	MS,Std,RI
2	Methyl thiolacetate	1056	1047	1.55	MS,Std,RI
3	Dimethyl disulfide	1089	1086	1.64	MS,Std,RI
4	Methyl isopropyl disulphide	1185	1192	1.85	MS,Std,RI
5	*S*-Methyl thiobutanoate	1207	1215	1.82	MS,Std,RI
6	Thiazole, 2-methyl-	1254	1250	1.55	MS,Std,RI
7	Thiazole	1267	1265	1.44	MS,Std,RI
8	4-Methylthiazole	1300	1279	1.52	MS,Std,RI
9	Ethyl methanesulfinate	1346		1.51	MS,Std,RI
10	Dimethyl trisulfide	1414	1378	1.84	MS,Std,RI
11	3-Mercapto-2-butanone	1582	1282	1.41	MS,Std,RI
12	Dimethyl sulfoxide	1593	1582	1.44	MS,Std,RI
13	3-Thiophenecarboxaldehyde	1725	1693	1.50	MS,Std,RI

14	S-Methyl 2-thiolfuroate	1882		1.58	MS,Std,RI
15	Dimethyl sulfone	1938	1912	1.33	MS,Std,RI
16	Benzothiazole	2001	1991	1.65	MS,Std,RI
17	Thiophene, tetrahydro-, 1,1-dioxide	2280		1.50	MS,Std
18	Methylpyrazine	1285	1276	1.58	MS,Std,RI
19	2,5-Dimethylpyrazine	1339	1333	1.68	MS,Std,RI
20	2,6-DimethylPyrazine	1348	1339	1.67	MS,Std,RI
21	Ethylpyrazine	1356	1344	1.69	MS,Std,RI
22	2,3-Dimethylpyrazine	1367	1357	1.68	MS,Std,RI
23	2-Ethyl-6-methylpyrazine	1406	1395	1.79	MS,Std,RI
24	2-Ethyl-5-methylpyrazine	1411	1402	1.76	MS,Std,RI
25	2-Methyl-3-isopropylpyrazine	1411	1387	1.88	MS,Std,RI
26	Trimethylpyrazine	1424	1408	1.76	MS,Std,RI
27	2,6-Diethylpyrazine	1454	1444	1.88	MS,Std,RI
28	3-Ethyl-2,5-dimethylpyrazine	1462	1450	1.88	MS,Std,RI
29	2,3-Dimethyl-5-ethylpyrazine	1480	1460	1.86	MS,Std,RI
30	Tetramethylpyrazine	1492	1460	1.85	MS,Std,RI
31	2,3,5-Trimethyl-6-ethylpyrazine	1529	1491	1.96	MS,Std,RI
32	*p*-Cymene	1322	1288	2.02	MS,Std,RI
33	Prenol	1336	1320	1.39	MS,Std,RI
34	Perillaldehyde	1353		2.09	MS,Std
35	3,4-Dimethylcumene	1389	1372	2.05	MS,RI,Tent
36	8-Hydroxy-7-methoxycoumarin	1392		2.05	MS,RI,Tent
37	(*Z*)-Linalool oxide	1457	1455	1.71	MS,Std,RI
38	(*E*)-Linalool oxide	1462	1471	1.74	MS,Std,RI
39	1,3,8-p-Menthatriene	1462		2.01	MS,Tent
40	Linalool	1556	1550	1.62	MS,Std,RI
41	Isophorone	1624	1621	1.77	MS,Std,RI
42	Terpineol	1713	1705	1.65	MS,Std,RI
43	4-Ketoisophotone	1722	1717	1.67	MS,Std,RI
44	*β*-Damascenone	1851	1838	1.93	MS,Std,RI
45	Geranylacetone	1873	1865	1.93	MS,Std,RI
Acetals					
1	1-Ethoxy-1-methoxyethane	895		1.57	MS,Tent
2	Diethoxymethane	897		1.61	MS,Std,RI
3	1,1-Diethoxyethane	923	900	2.23	MS,Std,RI
4	1,1-Diethoxypropane	951	950	1.98	MS,Std,RI
5	1-(1-Ethoxyethoxy)propane	962		2.00	MS,Tent

6	1,1-Diethoxybutane	967	988	2.09	MS,Std,RI
7	1,1-Diethoxy-2-methylpropane	969	969	2.14	MS,Std,RI
8	Trimethoxymethane	994		1.55	MS,Std
9	1-(1-Ethoxyethoxy)butane	1040		1.18	MS,Tent
10	1,1-Diethoxy-3-methylbutane	1090	1074	2.22	MS,Std,RI
11	1-(1-Ethoxyethoxy)propane	1124	1104	2.13	MS,Std,RI
12	1,1-Diethoxypentane	1148	1135	2.33	MS,Std,RI
13	1,1-Diethoxyhexane	1278	1242	2.50	MS,Std,RI
14	1-(1-Ethoxyethoxy)hexane	1280	1258	2.38	MS,Std,RI
15	1,1,3-Triethoxypropane	1320	1303	2.00	MS,Std,RI
16	1,1,3,3-Tetraethoxypropane	1795		1.21	MS,Tent
17	1,1-Diethoxyoctane	1439	1431	2.35	MS,Std,RI
18	1,1-Diethoxynonane	1532	1511	2.38	MS,Std,RI
19	Ethoxyethene	1934		1.54	MS,Std
Acids					
1	(*R*)-3-Hydroxybutyric acid	1439		1.42	MS,Std,RI
2	Acetic acid	1467	1452	1.20	MS,Std,RI
3	Propanoic acid	1553	1548	1.23	MS,Std,RI
4	2-Methylpropanoic acid	1580	1581	1.29	MS,Std,RI
5	2,2-Dimethylpropanoic acid	1590	1579	1.25	MS,Tent
6	Butanoic acid	1635	1637	1.30	MS,Std,RI
7	4-Hydroxybutanoic acid	1663		1.55	MS,Tent
8	isopentanoic acid	1681	1680	1.33	MS,Std,RI
9	2-Methylbutanoic acid	1684	1687	1.39	MS,Std,RI
10	Pentanoic acid	1721	1733	1.31	MS,Std,RI
11	2-Methylpentanoic acid	1779	1764	1.31	MS,Std,RI
12	Isocrotonic acid	1791		1.25	MS,Tent
13	isohexanoic acid	1819	1817	1.31	MS,Std,RI
14	Hexanoic acid	1863	1863	0.78	MS,Std,RI
15	2-Ethylhexanoic acid	1961	1965	1.30	MS,Std,RI
16	Heptanoic acid	1971	1971	1.38	MS,Std,RI
17	Octanoic acid	2077	2067	1.4	MS,Std,RI
18	Nonanoic acid	2181	2174	1.34	MS,Std,RI
19	Decanoic acid	2269	2270	2.33	MS,Std,RI
20	Undecanoic acid	2374	2371	1.92	MS,Std,RI
21	n-Decanoic acid	2287	2281	1.38	MS,Std,RI
22	(7Z)-7-Tetradecenoic acid	2326		1.80	MS,Tent
23	Tridecanoic acid	2582	2570	1.92	MS,Std,RI

24	Hydrocinnamic acid	2600	2603	1.73	MS,Std,RI
25	Tetradecanoic acid	2690	2692	1.99	MS,Std,RI
26	*n*-Hexadecanoic acid	2923	2923	2.74	MS,Std,RI
27	Octadecanoic acid	3088	3090	3.06	MS,Std,RI
28	Linoleic acid	3156	3168	2.94	MS,Std,RI
29	Oleic acid	3180	3184	2.29	MS,Std,RI

[a] RI of compound in DB-WAXext (RI); [b] RI in published literature with same parameter or similar column (RIL). [c] Identification: tentative identification (Tent) based on MS (compounds were identified by MS spectra) and RI (compounds were identified with the RI from the literatures); positive identification based on Std (compounds were identified by a comparison to the retention index and mass spectrum of authentic chemical standard).

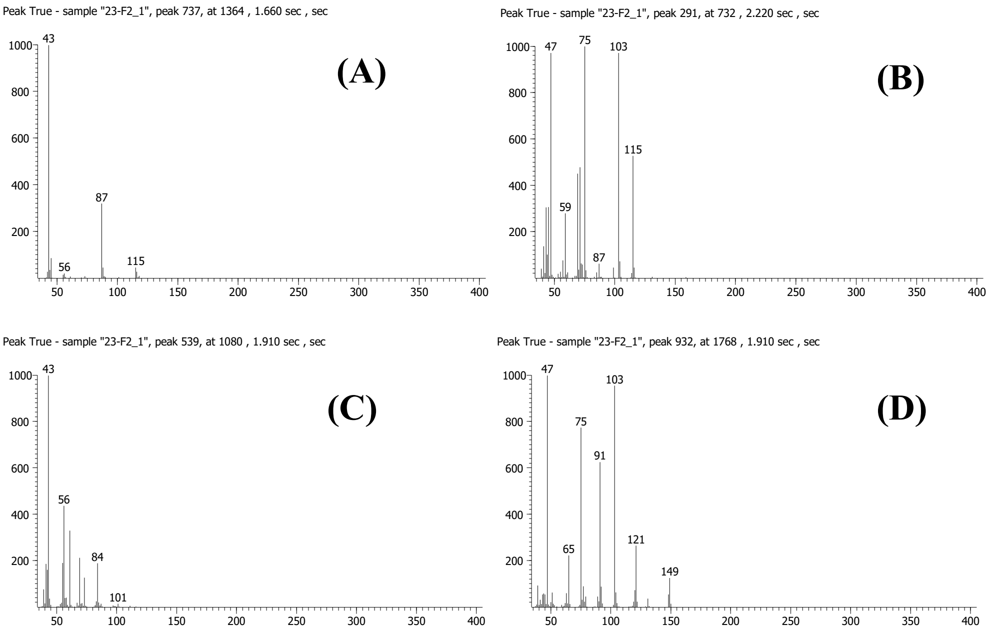

Figure S1 MS spectra of (A) ethyl hydrogen malonate; (B) 1,1-diethoxy-3-methyl-butane; (C) hexyl acetate; (D) benzeneacetaldehyde.
